# Cuminaldehyde from *Cinnamomum verum* Induces Cell Death through Targeting Topoisomerase 1 and 2 in Human Colorectal Adenocarcinoma COLO 205 Cells

**DOI:** 10.3390/nu8060318

**Published:** 2016-05-24

**Authors:** Kuen-daw Tsai, Yi-Heng Liu, Ta-Wei Chen, Shu-Mei Yang, Ho-Yiu Wong, Jonathan Cherng, Kuo-Shen Chou, Jaw-Ming Cherng

**Affiliations:** 1 Department of Internal Medicine, China Medical University Beigang Hospital, Yunlin 65152, Taiwan; d4295@yahoo.com.tw (K.-d.T.); yeeheng6061@gmail.com (Y.-H.L.); slowfish1234@yahoo.com.tw (T.-W.C.); kd2624@yahoo.com.tw (S.-M.Y.); pk1977wong@gmail.com (H.-Y.W.); 2 School of Chinese Medicine, College of Chinese Medicine, China Medical University, Taichung 40402, Taiwan; 3 Institute of Molecular Biology, National Chung Cheng University, Chiayi 62102, Taiwan; 4 Faculty of Medicine, Medical University of Lublin, Lublin 20-059, Poland; jcherngca@yahoo.com.tw; 5 Department of Family Medicine, Saint Mary’s Hospital Luodong, Yilan 26546, Taiwan; smh01062@smh.org.tw; 6 Department of Internal Medicine, Saint Mary’s Hospital Luodong, Yilan 26546, Taiwan; 7 St. Mary’s Junior College of Medicine, Nursing and Management, Yilan 26644, Taiwan

**Keywords:** cuminaldehyde, antiproliferative, topoisomerase I, topoisomerase II, lysosomal vacuolation, xenograft

## Abstract

*Cinnamomum verum*, also called true cinnamon tree, is employed to make the seasoning cinnamon. Furthermore, the plant has been used as a traditional Chinese herbal medication. We explored the anticancer effect of cuminaldehyde, an ingredient of the cortex of the plant, as well as the molecular biomarkers associated with carcinogenesis in human colorectal adenocarcinoma COLO 205 cells. The results show that cuminaldehyde suppressed growth and induced apoptosis, as proved by depletion of the mitochondrial membrane potential, activation of both caspase-3 and -9, and morphological features of apoptosis. Moreover, cuminaldehyde also led to lysosomal vacuolation with an upregulated volume of acidic compartment and cytotoxicity, together with inhibitions of both topoisomerase I and II activities. Additional study shows that the anticancer activity of cuminaldehyde was observed in the model of nude mice. Our results suggest that the anticancer activity of cuminaldehyde *in vitro* involved the suppression of cell proliferative markers, topoisomerase I as well as II, together with increase of pro-apoptotic molecules, associated with upregulated lysosomal vacuolation. On the other hand, *in vivo*, cuminaldehyde diminished the tumor burden that would have a significant clinical impact. Furthermore, similar effects were observed in other tested cell lines. In short, our data suggest that cuminaldehyde could be a drug for chemopreventive or anticancer therapy.

## 1. Introduction

Colorectal cancer is one of the most common malignancies [1]. Nevertheless, it is not sensitive to conventional chemotherapeutic drugs and there is a need for better management of the disease.

Over the past three decades, various approaches have been used for prevention and treatment of cancer, such as traditional Chinese medicine (TMC). The therapeutic usage described in classic books of Chinese materia medica are still informative even the present-day; for example, *Artemisia annua*. Artemisinin, an ingredient of the plant, was discovered by Tu Youyou, a Chinese scientist, who was awarded half of the 2015 Nobel Prize in Medicine for her discovery of its effect against *Plasmodium falciparum* malaria.

Moreover, contemporary epidemiological and experimental studies have unremittingly suggested a correlation between regularly eating vegetables and fruits and avoidance of lifestyle disorders, including tumors and heart disorders [2,3]. Phytochemicals, e.g., flavonoids and polyphenols of which plants are rich sources, appear to possess desirable characters required for avoiding malignancy and may have great possibility as antiproliferative drugs [4,5,6,7,8,9]. Indeed, the common seasoning cinnamon is manufactured from the true cinnamon tree. In addition, the plant has been applied for the treatment of dyspepsia, circulatory disorders, and inflammation, such as gastroenteritis [2,3]. Cuminaldehyde, an ingredient of true cinnamon tree’s bark, may be the compound that has this effect. Cuminaldehyde exists in the true cinnamon tree in a high concentration, and it is also found in the shoot of *Artemisia salsoloides*, leaf of *Aegle marmelos,* and essential oil from cumin [10]. The chemical is stable, soluble in ethanol, and available commercially. Until now, very little research on cuminaldehyde has been published. Therefore, the current study intended to explore the anticancer activity of cuminaldehyde and clarify its mechanisms in human colorectal adenocarcinoma COLO 205 cells.

Malignancy is a hyperproliferative disease. Various genetic and epigenetic aberrations are needed to convert normal cells into transformed ones. These abnormalities regulate different pathways which collaborate to enable malignant cells endowed with an extensive capabilities needed for proliferating, metastating, and killing their host [11]. Although antiproliferative drugs are possibly able to act through various mechanisms, apoptosis has been shown to be the most common and preferred mechanism through which many anticancer agents kill and eradicate cancer cells [12].

Apoptosis-inducing antiproliferative agents may act by targeting mitochondria. The drugs may alter mitochondria through various mechanisms. They may cause the development of pores on membranes, leading to swelling of mitochondria, or increase membrane permeability, resulting in the discharge of pro-apoptotic cytochrome from the organelle into the cytosolic compartment. Cytochrome *c* interacts with protease activating factor-1 together with deoxyadenosine triphosphate, which then interacts with pro-caspase-9 resulting in the formation of apoptosome. Then the inactive pro-caspase-9 is activated by the formed apoptosome into active caspase-9. Next, the active form caspase-9 acuates caspase-3, resulting in a proteolytic cascade [13,14,15].

Topoisomerases, enzymes controlling the DNA’s topological status, are involved in conserving the integrity of the genome [16]. They relax intertwined DNA by transitory protein-linked breaks of only one (topoisomerase I) or two (topoisomerase II) strands of the double-stranded DNA [17]. Topoisomerase I plays a role in DNA processing by engaging systems of tracking and being involved in conserving the integrity of the genome [16]. Upregulated enzyme’s catalytic activity, protein, and mRNA have been demonstrated across human cancers [18]. Indeed, topoisomerase I is involved in the chromosomal instability of colorectal cancer (CRC) and the expression levels of the enzyme has been suggested as prognostic markers [19,20,21] in CRC. Topoisomerase II is upregulated during cell growth and peaks at G2/M. Topoisomerase II gene copy number is also elevated in CRC and considered as a potential predictive biomarker for anticancer treatment [20]. In addition to cell cycle regulation, the enzyme has been demonstrated to be another main target of antiproliferative agents [22,23,24,25]. What is more, apoptotic cell death was shown to be the ultimate effective pathway of death in cancer subsequent to suppression of topoisomerase [26].

This diversification of machineries of carcinogenesis implies that there could be various processes that are crucially objective for avoidance of cancer. In an effort to investigate the activities and latent machineries of cuminaldehyde in human colorectal adenocarcinoma COLO 205 cell, we performed a series of tests to study the effects of cuminaldehyde on growth as well as activities of topoisomerase I and II in human colorectal COLO 205 cells. Our results prove that cuminaldehyde suppressed the activities of both topoisomerase I and II and increased lysosomal vacuolation with upregulated volume of acidic compartment together with cytotoxicity. Lastly, cuminaldehyde induced apoptosis, resulting in the suppression of cell proliferation, *in vitro* as well as *in vivo*.

## 2. Materials and Methods

### 2.1. Materials

We purchased RPMI-1640 and fetal bovine serum (FBS) from GIBCO BRL (Gaithersburg, MD, USA), together with dimethyl sulfoxide and cuminaldehyde from Sigma-Aldrich, Inc. (St. Louis, MO, USA).

### 2.2. Cell Culture

Human colorectal adenocarcinoma COLO 205 cells (American Type Culture Collection CCL-222, American Type Culture Collection, Manassas, VA, USA) were purchased via BCRC (Bioresource Collection and Research Center, Hsinchu, Taiwan) on 27 July 2010 and stored in liquid nitrogen until usage. The cells were incubated in the medium of RPMI-1640, complemented with penicillin 10 U/mL, amphotericin B 0.25 μg/mL, streptomycin 10 μg/mL, and FBS 10% (*v/v*) at 37 °C with 5% carbon dioxide.

### 2.3. Cell Viability XTT Test

We incubated the cells in the culture plate with 96 wells at the concentration of ten thousand cells per well. After being incubated for 24 h, we treated the cells with cuminaldehyde at the concentration of 10, 20, 40, 80, or 160 µM for 12, 24, or 48 h. We determined cell viability using the Cell Proliferation Kit II (XTT) (Roche Applied Science, Mannheim, Germany) according to the supplier’s instructions. The value of absorbance was evaluated by a spectrophotometer (Tecan infinite M200, Tecan, Männedorf, Switzerland) using 492 nm wavelength with a reference of 650 nm wavelength.

### 2.4. Lactate Dehydrogenase Cytotoxicity Test

We incubated the cells in the culture plate with 96 wells at the concentration of ten thousand cells per well. After being incubated for 24 h, cells were incubated with various cuminaldehyde’s concentrations for 48 h. Lactate dehydrogenase’s activity was evaluated by LDH-Cytotoxicity Kit (BioVision, Milpitas, CA, USA) according to the supplier’s instructions. The samples’ absorbance at 490 nm wavelength was evaluated by a spectrophotometer (Tecan infinite M200, Tecan, Männedorf, Switzerland). Data are presented as the percent of activity’s variation relative to untreated control.

### 2.5. Test for Nuclear Fragmentation

Nuclear fragmentation test using acridine orange was performed to investigate the possible mechanism of suppressive effect of cuminaldehyde on growth in human colorectal COLO 205 cells. We cultured the cells with various cuminaldehyde concentrations for 48 h and stained the cells with acridine orange (5 μg per mL) at 25 °C. The cells were then examined by the Nikon ECLIPSE T*i* fluorescence microscope [27].

### 2.6. Comet Test

Comet test is an electrophoretic assay and has been employed to study the injury of DNA in eukaryotic cells individually. The assay is comparatively easy to achieve, versatile, and sensitive. The sensitivity’ limit is approximately 50 strand breakages per diploid cell. This test was achieved following Olive’s alkaline protocol (with 4′,6-diamidino-2-phenylindole staining) [28]. The cells were then observed using the Nikon ECLIPSE T*i* fluorescence microscope with C-FL Epi-Fl Filter Cube and analyzed with automated analytical software (Comet Assay 2.0, Perceptive Instruments, Bury St. Edmunds, UK) following the manufacturer’s instructions.

### 2.7. Test for Volume of Acidic Compartments

Increase of the volume of acidic compartment is a general phenomenon of the cells subjected to necrotic or apoptotic death of the cell. Moreover, upregulated volume of the acidic compartment may be an implication of cells that are about to die [29]. To investigate the activities of cuminaldehyde in the cell, the volume of acidic compartment test for lysosomes was achieved as reported formerly [27] with modification. Briefly, human colorectal COLO 205 cells were seeded in 6 cm dishes at the density of 6000/cm^2^ (instead of 6250/cm^2^) 24 h before cuminaldehyde was added. After incubation with cuminaldehyde for another 48 h (instead of 24 h), the cells were washed twice with PBS (phosphate-buffered saline) and incubated for 4 min with 4 mL staining solution. The rest of the experiment was performed similarly. The optical density (OD) at 540 nm of samples was determined by a spectrophotometer (Tecan infinite M200, Tecan, Männedorf, Switzerland). All tests were performed in triplicate.

### 2.8. Mitochondrial Membrane Potential Test

Mitochondrial dysfunction plays a crucial role in the initiation of apoptotic cell death. Actually, the opening of the transition pore creates depolarization of the mitochondrial membrane potential and releasing of apoptogenic factors [30]. To investigate the mitochondria’s role of in cuminaldehyde-caused apoptotic cell death, we observed the variations in mitochondrial membrane potential.

The potential of mitochondrial membrane was determined by the reagent JC-1, a mitochondrial-specific fluorescent compound (Invitrogen, Carlsbad, CA, USA.) according to the protocol described previously [31]. The JC-1 reagent is monomer and the mitochondrial membrane potential is less than 120 millivolt. Under such a condition, the dye emits green fluorescence (wavelength of 540 nm) after excitement by blue light (wavelength of 490 nm). In addition, the dye becomes dimmer (J-aggregate) at a mitochondrial membrane potential of more than 180 millivolt and emits red fluorescence (590 nm) after excitation by green light (540 nm). Human colorectal COLO 205 cells were treated with various cuminaldehyde concentrations for 48 h, harvested, and then stained with JC-1 at the concentration of 25 μM at 37 °C for 30 min. Finally, the samples were examined using a spectrophotometer and a fluorescence microscope. Changes in the percentage of red (wavelength of 590-nm)/green (wavelength of 540-nm) fluorescence represent the variations of membrane potential [32].

### 2.9. Caspase Activity Test

Proteins of mitochondrial called SMACs (second mitochondria-derived activator of caspases) are discharged into the cytosolic compartment after the increased membranes’ permeability. Then, SMAC interacts with the inhibitor of apoptosis proteins (IAPs), thereby making IAPs inactive, which then abolishes IAPs from inhibiting caspases [33,34] that demolish the cell subsequently.

To farther explore the details in cuminaldehyde-caused apoptotic cell death, the variations in activities of the crucial caspases implicated in apoptotic cell death were determined. The assay is established on the evaluation of the AFC chromophore following division from DEVD- and LEHD-AFC through caspase-3 and -9, respectively. The released AFC emits a yellow-green fluorescence. Human colorectal COLO 205 cells were treated with various concentrations of cuminaldehyde for 48 h and activities of the caspases were measured by Fluorometric Assay Kit (BioVision, Milpitas, CA, USA) according to the supplier’s instructions. The light emission was determined by a spectrophotometer (Tecan infinite M200, Tecan, Männedorf, Switzerland). Data are presented as the percent of activity’s variation relative to control.

### 2.10. Test for Topoisomerase I and II Activities

Topoisomerase I and II extracts from the cells were prepared according to the methods of TopoGEN (Port Orange, FL, USA). Briefly, cells from two 100 mm dishes were pelleted at 800× *g* for 3 min at 4 °C, resuspended in 3 ml of ice cold TEMP buffer (10 mM Tris-HCl, pH 7.5, 1 mM EDTA, 4 mM MgCl_2_, 0.5 mM PMSF). The sample was then pelleted as described above, resuspended in 3 mL of TEMP and kept on ice for 10 min. The sample was then homogenized using tight fitting homogenizer with eight strokes. Nuclei were pelleted by centrifugation at 1500× *g* for 10 min at 4 °C, resuspended in 1 mL of cold TEMP, transferred to a microfuge tube, and pelleted in Microfuge at 1500× *g* at 4 °C for 2 min, sequentially. The nuclear pellet was resuspended in a small volume (no more than 4 pellet volumes) of TEP (same as TEMP but lacking MgCl_2_). An equal volume of 1 M NaCl was added. The sample was then vortexed, kept on ice for 60 min, and centrifuged at 100,000× *g* for 1 h at 4 °C. Tests for topoisomerase I as well as II activities were performed according to the methods described by Har-Vardi *et al.* [35].

### 2.11. In Vivo Tumor Xenograft Study

The study has been approved by the Institutional Animal Care and Use Committee (IACUC) of China Medical University that conforms to the provisions of the Declaration of Helsinki (the animal ethical approval number: 97-108-N). Nude mice (male, 6 weeks old, BALB/c Nude) were from the National Science Council Animal Center (Taipei City, Taiwan, Republic of China). The animals were raised under pathogen-free conditions under China Medical University’s regulations and ethical guidelines for the use and care of laboratory animals. Human colorectal COLO 205 cells (5 × 10^6^ cells in 200 μL of culture medium) were subcutaneously injected into the mice’s flanks. Treatment was started when the tumors reached about 75 mm^3^. Thirty-two mice were divided randomly into four groups (eight mice/group). Cuminaldehyde-treated mice received intratumoral injection of 5, 10, or 20 mg/kg/day of cuminaldehyde in a 200 μL volume (the solutions were prepared from stock solution of cuminaldehyde in dimethyl sulfoxide and diluted into appropriate concentrations in PBS) daily. The mice in the control group were treated with an equal volume of vehicle. After transplantation, body weight as well as tumor size were monitored at weekly intervals. Tumor size was measured using calipers, and tumor volume was calculated using the hemiellipsoid model formula (1):

tumor volume = 1*/*2(4*π/*3) × (*l/*2) × (*w/*2) × *h* (1)

where *l* = length, *w* = width, and *h* = height.

Specimens (tumor masses) at the end of the experiment (42 days after the treatment) were investigated by terminal deoxynucleotidyl transferase dUTP nick end labeling test using the Quick Apoptotic DNA Ladder Detection Kit (Chemicon, Temecuba, CA, USA) according to the supplier’s instructions.

### 2.12. Statistical Analysis

Results are presented by means plus/minus standard error. The statistical significance was determined by ANOVA (one-way analysis of variance), followed by the Bonferroni *t*-test for multiple comparisons. A *p* value lower than 0.05 was regarded as statistically significant.

## 3. Results

### 3.1. Cuminaldehyde’s Effects on Cell Morphological Changes

**Figure 1:**
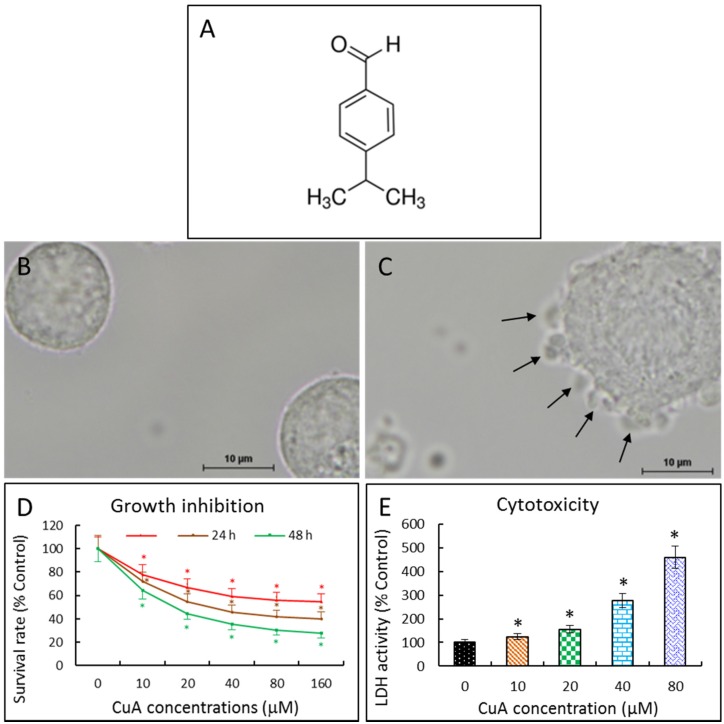
Cuminaldehyde’s chemical structure and effects on cellular morphology, proliferation, as well as lactate dehydrogenase releasing in human colorectal COLO 205 cells. (**A**) Chemical structure; (**B**) and (**C**) Cuminaldehyde’s effect on cellular morphology; Cells were treated without (**B**) and with 20 μM (**C**) cuminaldehyde for 48 h. Cell detachment, shrinkage, and blebbing of plasma membrane (arrows) were found when the cells were incubated with 20 μM of cuminaldehyde; (**D**) Cuminaldehyde’s effect on growth. Human colorectal COLO 205 cells were treated with cuminaldehyde at the specified circumstances. Proliferation suppressive effect was determined using the XTT test; (**E**) Cuminaldehyde’s effect on the lactate dehydrogenase releasing in the cells. The supernatant was gathered after 48 h of incubation with the indicated cuminaldehyde concentrations. Absorptions of light were determined by a spectrophotometer (Tecan infinite M200, Tecan, Männedorf, Switzerland). Results are shown by means plus/minus standard error of the mean, *n* equal to 3. *, Statistically significant (*p* less than 0.05) from the control group. CuA, cuminaldehyde.

When human colorectal COLO 205 cells were incubated with 20 μM of cuminaldehyde for 48 h, blebbing of the plasma membrane was found. In addition, cell shrinkage and cell detachment also occurred (Figure 1C).

### 3.2. Cuminaldehyde Inhibited Human Colorectal COLO 205 Cell Proliferation

Different methods have been used for quantifying cell growth; for instance, DNA synthesis as well as metabolic activity. Although radioactive labelling of synthesized DNA is the most accurate assay for DNA quantification, the disadvantages of this assay are the hazards and hassle of using radioactivity. An alternative method quantifying growth is the metabolic activity. The assay is established on the cleavage of a salt (the tetrazolium, e.g., XTT and MTT) into formazan by cell’s dehydrogenases which then modifies culture medium’s color. This assay is easier, faster, and does not require the use of radioactive materials.

We investigated cuminaldehyde’s potential cell proliferation inhibitory activity in human colorectal COLO 205 cells by the XTT test. As demonstrated in Figure 1D, cuminaldehyde inhibited cell proliferation in a dose- as well as time-dependent manner. The IC_50_ value following 48 h of treatment was 16.31 μM.

### 3.3. Cuminaldehyde Caused Cytotoxicity in Human Colorectal COLO 205 Cells

The first morphological evidence of apoptotic phenomenon is retraction of the cell, loss of adherence, followed by convolution of cytoplasm and membrane of the plasma, together with blebbing. Finally, the cell disintegrated into small particles called apoptotic bodies, leading to the release of the cell’s content into the bathing medium [36]. One way of studying loss of integrity of the membrane is determining the releasing of enzyme lactate dehydrogenase into the supernatant medium [37]. The assay was initially employed to test cellular death developed through necrosis [38]. Then, the assay was shown to accurately quantify apoptosis [39,40,41].

Cuminaldehyde was cytotoxic, as proved by the elevation of lactate dehydrogenase activity in the bathing medium (Figure 1E).

### 3.4. Cuminaldehyde Caused Nuclear Fragmentation in Human Colorectal COLO 205 Cells

Apoptosis is the most frequent and preferred mechanism through which various anticancer drugs kill cancer cells [12]. Moreover, apoptosis also has been shown to be the major machinery of the death of cancer caused by several polyphenols [42,43,44,45]. In the nucleus, apoptosis is characterized by endonuclease activation, resulting in cleavage of nucleic acid into fragments.

Acridine orange is a dye with nucleic acid-selective metachromatic characteristic and valuable for quantifying apoptosis, determinations of cell cycle, proton-pump activity, and pH gradients [46]. When acridine orange inserts into double-stranded DNA, it fluoresces green. In addition, when interacting with RNA or single-stranded DNA, acridine orange fluoresces orange. Apoptotic cells which contain a high fraction of the nucleic acid in the denaturated status exhibit an orange fluorescence along with a diminished green one relative to interphase non-apoptotic cells. In addition, when acridine orange are in an acidic environment (e.g., cellular lysosomes), the dye becomes protonated as well as sequestered. Under such an acidic environment, when excited by the blue light, the dye fluoresces orange [47]. The test of nuclear fragmentation is established on acridine orange’s characters and examined microscopically.

**Figure 2:**
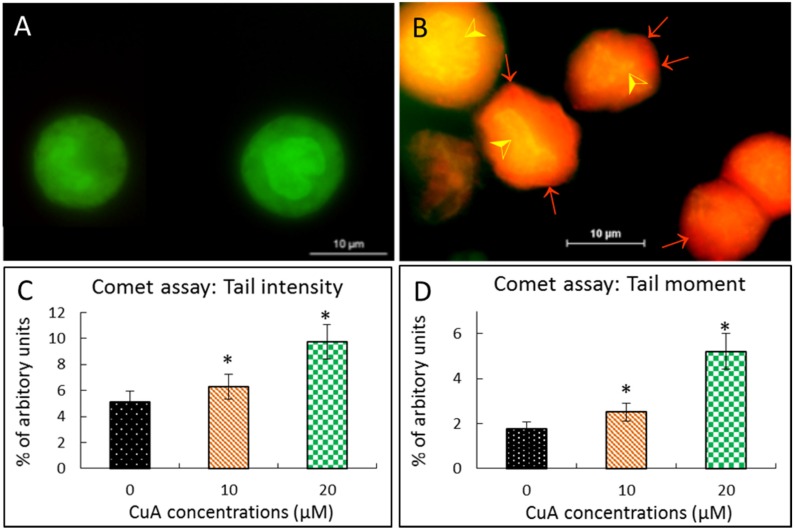
Cuminaldehyde caused nuclear fragmentation in human colorectal COLO 205 cells. (**A** and **B**) Acridine orange staining; COLO 205 cells were incubated without (**A**) with 20 μM (**B**) cuminaldehyde, respectively, for 48 h, then stained using acridine orange. The orange vacuoles in COLO cells demonstrate that they existed acidic; (**A**) Typical picture of control cells accompanying intact nucleus with green fluorescence that implicates a good cell viability; (**B**) Typical picture of test cells incubated with cuminaldehyde with lysosomal vacuolation (arrows) and nuclear fragmentation (arrow heads) were found; (**C** and **D**) Comet test. Cuminaldehyde’s effect on intensities of tail (**C**) as well as moment (**D**). Human colorectal COLO 205 cells were incubated with cuminaldehyde at the indicated concentrations for 48 h. Data are shown as means plus/minus standard error of the mean, *n* = 125. *, Significant difference (*p* < 0.05) from the control. CuA, cuminaldehyde.

When human colorectal COLO 205 cells were treated with cuminaldehyde at the concentration of 20 μM for 48 h, the result of staining using acridine orange demonstrated that COLO cells demised partially through apoptosis, along with fragmentation and nuclear condensation. In addition, orange-stained lysosomal vacuoles were observed. On the other hand, no significant chromosomal fragmentation was found in the control group (Figure 2A).

DNA strand breakage was also explored using the comet test after treatment with various cuminaldehyde concentrations. As demonstrated in Figure 2C,D, treatment with cuminaldehyde led to increased tail intensity as well as moment.

Given that nuclear condensation, fragmentation, blebbing of the plasma membrane and the formation of apoptotic body are apoptosis’s morphologic characteristics [48], the morphological changes observed in the study prove that treatment with cuminaldehyde did lead to apoptosis in human colorectal COLO 205 cells (Figure 1C and Figure 2B).

### 3.5. Cuminaldehyde Increased Volume of Acidic Compartment in Human Colorectal COLO 205 Cells

**Figure 3:**
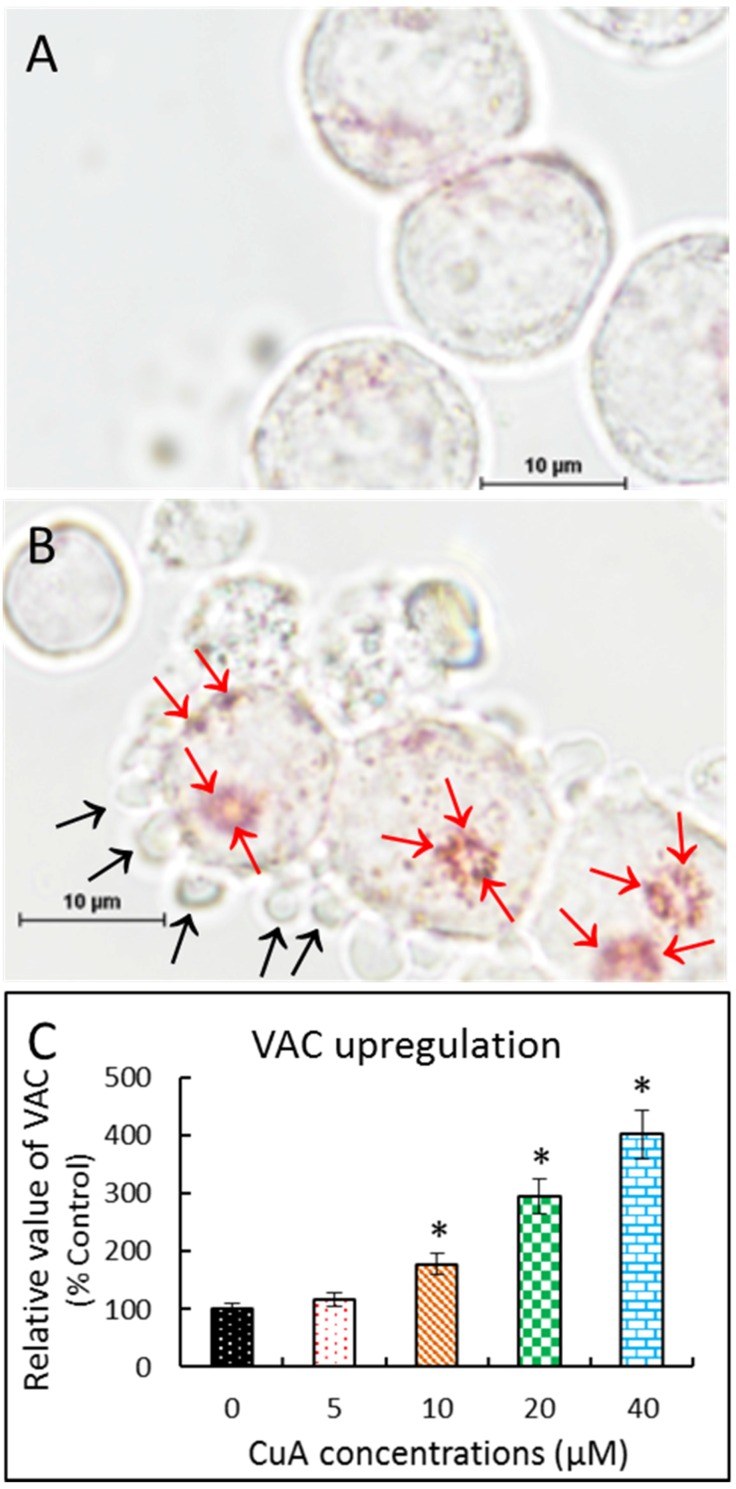
Cuminaldehyde increased the volume of the acidic compartment in human colorectal COLO 205 cells. After treatment without and with 20 μM cuminaldehyde, respectively, for 48 h, human colorectal COLO 205 cells were stained using neutral red. (**A**) Human colorectal COLO 205 cells without treatment: There were no observable vacuoles in the cell; (**B**) Human colorectal COLO 205 cells treated with cuminaldehyde at the concentration of 20 μM for 48 h. The blebbing (black arrows) and acidic red-stained vacuoles (red arrows) in cells happened; (**C**) Cuminaldehyde increased volume of acidic compartment in a quantity-dependent manner. After treating the cells using the specified concentrations of cuminaldehyde for 48 h, results were evaluated by a spectrophotometer. Results are shown by means plus/minus standard error of the mean, *n* equal to 3. *, Statistically significant (*p* less than 0.05) from the control group. CuA, cuminaldehyde.

Neutral Red has been used to stain lysosomes and quantify the volume of acidic compartment in cells [27,49,50]. As demonstrated in Figure 3A,B, positive neutral red staining suggests that incubation with cuminaldehyde resulted in acidic vacuoles in human colorectal COLO 205 cells. Moreover, Figure 3C shows that the treatment increased the volume of the acidic compartment in a quantity-dependent manner in the cells.

### 3.6. Cuminaldehyde Caused Apoptosis via the Mitochondrial Pathway in Human Colorectal COLO 205 Cells

**Figure 4:**
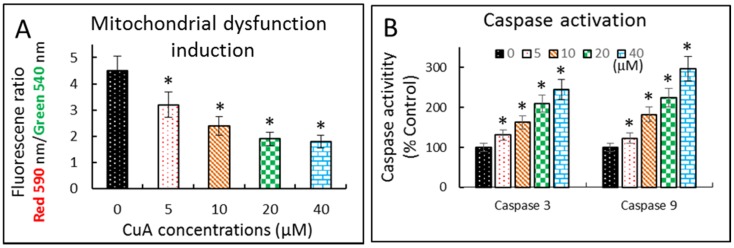
Cuminaldehyde caused apoptosis via the mitochondrial pathway in human colorectal COLO 205 cells. (**A**) Cells were treated with the specified cuminaldehyde concentrations for 48 h and mitochondrial membrane potential was evaluated using JC-1 spectrophotometrically; (**B**) Activations of caspase-3 as well as -9. After treating the cells using the specified concentrations of cuminaldehyde for 48 h, activities of caspases-3 and -9 were determined using a spectrophotometer. Results are expressed by means plus/minus standard error of the mean, *n* equal to 3. *, Statistically significant (*p* less than 0.05) from the control group. CuA, cuminaldehyde.

We then investigated the mitochondria’s role of in the cuminaldehyde-caused apoptosis in human colorectal COLO 205 cells. Initial apoptotic cell death frequently involves mitochondrial depolarization, followed by releasing of mitochondrial apoptogenic molecules into cytosol. Therefore, we explored mitochondrial dysfunction by determining mitochondrial membrane potential in cuminaldehyde-treated human colorectal COLO 205 cells using the mitochondria-specific dye JC-1 with a spectrophotometer. Figure 4A shows that cuminaldehyde caused the loss of mitochondrial membrane potential, as suggested by downregulation of mitochondrial membrane potential in a quantity-dependent manner.

Caspases are cysteine proteases that play critical roles in apoptosis. Figure 4B shows that the activities of caspase-3 and -9 elevated in a quantity-dependent manner in cuminaldehyde-treated human colorectal COLO 205 cells.

### 3.7. Cuminaldehyde Suppressed Topoisomerase I Activity in Human Colorectal COLO 205 Cells

**Figure 5:**
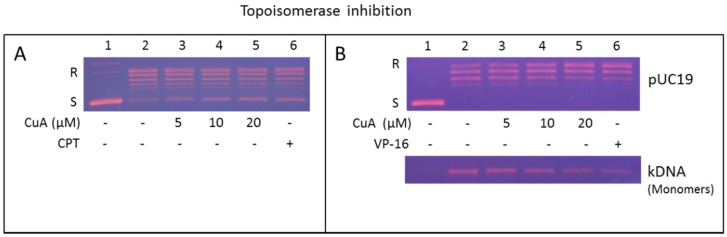
Cuminaldehyde inhibited topoisomerase I as well as II activities in human colorectal COLO 205 cells. (**A**) Cuminaldehyde inhibited topoisomerase I activity. Nuclear proteins of COLO 205 cells interacted with the indicated cuminaldehyde concentrations in a topoisomerase I’s specific reaction mixture (lanes 3–5), or 60 μM of camptothecin (CPT, a specific topoisomerase I inhibitor and used as positive control, lane 6), or the vehicle (1% dimethyl sulfoxide, lane 2). Lane 1, pUC19 DNA only; (**B**) Cuminaldehyde inhibited topoisomerase II activity. DNA relaxation test (upper panel) and decatenation test (lower panel). Nuclear proteins of COLO 205 cells were added to a specific topoisomerase II reaction mixture with the specified cuminaldehyde concentrations (lanes 3–5) or 60 μM of camptothecin (a specific suppressor of topoisomerase II and used as positive control, lane 6), or the vehicle (one percent dimethyl sulfoxide, lane 2). Lane 1, Interwined pUC19 DNA (upper panel) or kinetoplast DNA (lower panel) only. kinetoplast DNA is an extensive chain of plasmids. When kinetoplast DNA is examined using electrophoretic analysis, it gets the gel only a lightly (figure not demonstrated). Consequent to topoisomerase II’s decatenation, small monomeric circles of nucleic acid were produced (lower panel, lane 2–6). This is the representative of six experiments. CPT, camptothecin; CuA, cuminaldehyde; kDNA, kinetoplast; S & R, Interwined and the unrestrained forms of the pUC 19 plasmid, respectively; VP-16, etoposide.

The effect of cuminaldehyde on activity of topoisomerase I in human colorectal COLO 205 cells was performed with increasing cuminaldehyde concentration (Figure 5A, lane 3–5) or camptothecin (a known specific suppressor of type I topoisomerase and used as a positive control, lane 6) [51]. Figure 5A shows the transformation of the intertwined plasmid pUC 19 into the unrestrained form declined in a quantity-dependent manner under the existence of cuminaldehyde or camptothecin (please correlate lane 3–6 to lane 2). These data suggest that cuminaldehyde suppressed the DNA loosening activity topoisomerase I the human colorectal COLO 205 cell nuclear proteins.

### 3.8. Cuminaldehyde Suppressed Activity of Topoisomerase II in Human Colorectal COLO 205 Cells

The effect of cuminaldehyde on topoisomerase II activity in human colorectal COLO 205 cells was investigated using increasing concentration of cuminaldehyde (Figure 5B, lane 3–5) or etoposide (a known inhibitor of topoisomerase II and used as a positive control, lane 6) [52]. Figure 5B, upper panel, shows transformation of the interwined plasmid pUC 19 into the unrestrained form declined in a quantity-dependent manner under the existence of cuminaldehyde or etoposide (please correlate lane 3–6 to lane 2). The data suggest that cuminaldehyde suppressed DNA relaxation activity of topoisomerase II in the human colorectal COLO 205 cell nuclear proteins. In addition, this effect was further evaluated using the decatenation test. The decatenation effect involves the releasing of mini circular DNA (monomers) from the kinetoplast, an extensive chain of plasmids. Nuclear proteins in human colorectal COLO 205 cells enclosed type II topoisomerase that transformed kinetoplast to monomeric DNA (Figure 5B, lower panel, please correlate lane 2 to lane 1). The transformation of kinetoplast into monomeric DNA declined in a quantity-dependent manner under the existence of cuminaldehyde (please correlate lane 3–5 to lane 2) or etoposide (please correlate lane 6 to lane 2). The data suggest cuminaldehyde suppressed the topoisomerase II’s decatenation activity in the human colorectal COLO 205 cell nuclear proteins.

### 3.9. Cuminaldehyde Suppressed Growth of Human Colorectal COLO 205 Xenograft in a Nude Mice Model

**Figure 6:**
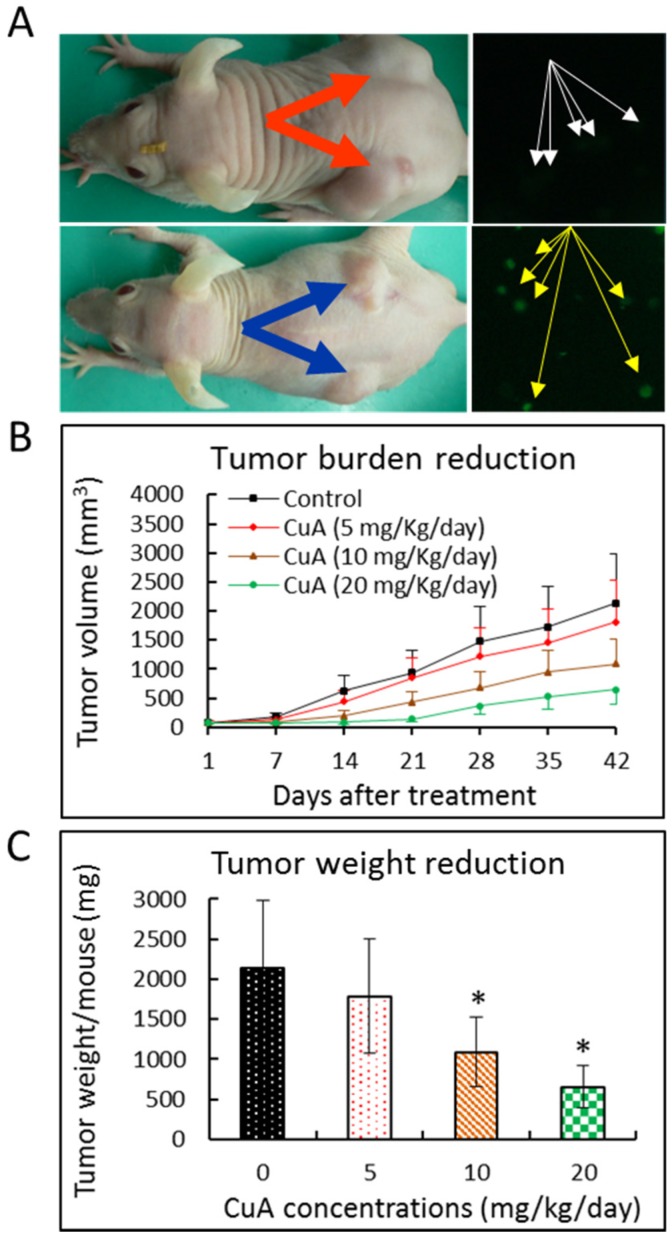
Cuminaldehyde suppressed growth and caused apoptosis in human colorectal COLO 205 xenograft. The mice with pre-established cancers (*n* = 8 per group) were treated using intratumoral injection with the specified cuminaldehyde concentrations. Tumor volumes were recorded by calipers and apoptosis was evaluated by terminal deoxynucleotidyl transferase dUTP nick end labeling test. (**A**) Left panel, Representative of tumor-bearing mice from the control (orange arrows) and 20 mg/kg/day of cuminaldehyde-injected (blue arrows) groups; (**A**) Right panel, cuminaldehyde caused apoptosis in human colorectal COLO 205 xenograft using terminal deoxynucleotidyl transferase dUTP nick end labeling test. Representative of terminal deoxynucleotidyl transferase dUTP nick end labeling test of tumors from the control (white arrows) and 20 mg/kg/day of cuminaldehyde-injected (yellow arrows) groups; (**B**) Mean of tumor volume observed at the specified number of days after the start of treatment; (**C**) Cuminaldehyde’s effects on tumor weight observed at the endpoint of the experiment. Tumor weight per mouse was collected and analyzed. Results are shown by means plus/minus standard error of the mean, *n* = 8. *, Statistically significant (*p* less than 0.05) from the control group. CuA, cuminaldehyde.

To investigate if cuminaldehyde suppresses proliferation of the human colorectal COLO 205 xenograft, 5 × 10^6^ human colorectal COLO 205 cells in 200 μL of culture medium were used for subcutaneous injection. Figure 6A, left panel, shows that, in comparison with tumors of control mice (orange arrows), obvious tumor burden reduction was found in the tumors of the mice injected with 20 mg/kg/day of cuminaldehyde (blue arrows). Tumor growth inhibition was found in all groups with cuminaldehyde injection (5, 10, and 20 mg/kg/day of cuminaldehyde, respectively). On the other hand, significant growth inhibition was observed only in mice injected with 10 and 20 mg/kg/day of cuminaldehyde, where about 48.9% and 69.4%, respectively, decreases in tumor volume were found (Figure 6B,C). None of the cuminaldehyde injections resulted in any significant decrease in body weight and/or diet consumption relative to the control group. The mechanism of cuminaldehyde’s antiproliferative effect *in vivo* was explored. We gathered the human colorectal COLO 205 xenograft from vehicle and cuminaldehyde-treated mice, then investigated the cause of the death by the terminal deoxynucleotidyl transferase dUTP nick end labeling assay. Figure 6A, right panel, demonstrates that, in comparison with tumors of control mice (white arrows), elevated terminal deoxynucleotidyl transferase dUTP nick end labeling-positive cells that suggest apoptotic death were found in the cancers of the cuminaldehyde-injected mice (yellow arrows).

## 4. Discussion

In addition to providing taste and flavor to foods, certain spices have been used as remedies in traditional medicine [53]. True cinnamon tree is used to manufacture the seasoning cinnamon and has been used for more than 5000 years by both of the two most ancient forms of medicine in the words: Ayurveda and traditional Chinese herbal medicines for various applications such as adenopathy, rheumatism, dermatosis, dyspepsia, stroke, tumors, elephantiasis, trichomonas, yeast, and virus infections [54]. Cuminaldehyde, an ingredient of the cortex of the plant, possesses various activities, including: (i) suppressions of melanin formation (through inhibiting the oxidation of l-3,4-dihydroxyphenylalanine catalyzed by tyrosinase) [55], lipoxygenase [56], aldose reductase, α-glucosidase [57], alpha-synuclein fibrillation (possibly by the interaction with amine groups through cuminaldehyde’s aldehyde group as a Schiff base reaction) [58]; and (ii) insulinotropic and β-cell protective action (through the closure of the ATP-sensitive K channel and the increase in intracellular Ca^2+^ concentration) [59].

Although cuminaldehyde exists in true cinnamon tree in a high concentration (100 PPM), it is also found in the shoot of *Artemisia salsoloides* (1000 PPM), leaf of *Aegle marmelos* (300 PPM), and essential oil from cumin [10]. Essential oil from cumin with the major constituents of cuminaldehyde, cymene, and terpenoids has been reported to possess: (i) antibacterial, antifungal, and insecticidal [60] activities; (ii) antioxidant capacity; (iii) anticancer activity [61,62] with glutathione-*S*-transferase activating, β-glucuronidase and mucinase inhibiting properties [60].

In this research, we initially explored the effects of cuminaldehyde on the proliferation of human colorectal COLO 205 cells. We observed that cuminaldehyde suppressed the growth of human colorectal COLO 205 cells in a concentration- as well as time-dependent manner (Figure 1D). Although cells may die through necrotic or other mechanisms, apoptosis is the preferred and most common mechanism through which different anticancer drugs kill as well as remove cancer cells [12]. Moreover, apoptosis was demonstrated to be the main machinery of tumor cell demise caused by several polyphenols [42,43,44,45].

Our data demonstrate that cuminaldehyde caused apoptotic cell death, as suggested by loss of mitochondrial membrane potential, increase of caspase-3 and -9 (Figure 4), along with morphological features of apoptosis, including apoptotic body formation, fragmentation, and nuclear condensation as demonstrated in different stainings as well as comet assay (Figure 1, Figure 2 and Figure 3).

Our data also suggest that cuminaldehyde generated vacuolation associated increased volume of the acidic compartment. Increase of volume of the acidic compartment has been demonstrated to be an ordinary event observed in cells that are subjected to apoptotic or necrotic cell demise and could be an indication of failing cells [29]. Because apoptotic cell death is an ordered process, an upregulated volume of acidic compartment could cause the self-digestion in the course of cell death [29].

In addition to cell cycle control, topoisomerase has been demonstrated to be another main target of anticancer drugs [22,23,24,25]. The chemotherapeutic agent etoposide kills tumor cells by stabilizing the transient intermediate division complex. The resulting accumulation of division complexes may lead to the development of permanent DNA strand divisions that fragment the chromosome leading to the stimulation of death pathways [63]. Furthermore, apoptosis has been demonstrated to be the most efficient death-pathway in cancer cells subsequent to the suppression of topoisomerase II [26]. Clinically, topoisomerase has been suggested as a potential predictive biomarker in CRC [20,64]. Topoisomerase I seems to be involved in the chromosomal instability pathway of sporadic CRC [21] and high frequency of gene gain of the topoisomerase I and II genes in CRC [20,65]. CRC patients with low topoisomerase I expression were statistically favorably associated with overall survival [19].

Our findings prove that cuminaldehyde inhibited activities of topoisomerase I and II in a quantity-dependent manner (Figure 5), which, in part, could be a machinery causing the cells to move toward apoptosis. Although most of inhibitors of topoisomerase are specifically targeting either type I or II topoisomerase [66], our results clearly show that cuminaldehyde inhibited activities of topoisomerase I along with II in human colorectal COLO 205 cells.

Our results clearly demonstrated that cuminaldehyde possesses antiproliferative activity in human colorectal COLO 205 cells. Furthermore, cuminaldehyde thiosemicarbazone has been shown to possess antiproliferative with anti-topoisomerase II activity in U937 cells [67]. However, some other tumor cell lines did not show the same negative effect of the plant extracts containing cuminaldehyde on cell proliferation [58]. Possible explanation for these contradictory phenomena could be the extracts also possess antioxidant [61] and/or other activities. Therefore, further research is needed to clarify the specific latent mechanisms of the suppression, possible mutagenic effects, as well as other side effects for clinical usage of cuminaldehyde as an anticancer and/or chemopreventive drug against human colorectal adenocarcinoma and/or other malignancies.

Treatment-associated cytotoxicity and other side effects of antiproliferative drugs are the main concerns of anticancer therapy. Consequently, the perfect anticancer agent would discriminatorily destroy malignant cells but not the healthy ones. Our results show that none of the therapy with cuminaldehyde caused any observable decline in body weight or consumption of diet relative to the control mice. Our data present persuasive evidence of the protecting activity of cuminaldehyde against human colorectal COLO 205 xenograft growth in the current study using nude mice model without any detectable side effect; this implies that cuminaldehyde has an antiproliferative effect in human colorectal COLO 205 cells and this agent may potentially serve as an anticancer and/or chemopreventive drug.

## 5. Conclusions

Collectively, our data clearly suggest that the antiproliferative effect of cuminaldehyde in human colorectal COLO 205 cells *in vitro* involved inhibition of cell growth markers, topoisomerase I and II, together with upregulation of proapoptotic molecules, associated with increased lysosomal vacuolation. *In vivo*, cuminaldehyde diminished the tumor burden and may have significant clinical impact.

The present study provides fundamental knowledge on the cancer inhibitory activity of cuminaldehyde in human colorectal COLO 205 cells that implicates a model for the exploration of possible antiproliferative drugs against human colorectal adenocarcinoma. Indeed, similar effects were observed in other tested cell lines, including human hepatocellular carcinoma SK-Hep-1 and Hep 3B, lung squamous cell carcinoma NCI-H520 and adenocarcinoma A549, and T-lymphoblastic MOLT-3. Our results present a rationalization for further developing cuminaldehyde as an effective and safe anticancer and/or chemopreventive drug. A future direction would be to synthesize the derivatives of cuminaldehyde and examine the protective effects of cuminaldehyde and their derivatives *in vitro*. We would then extend the study to examine the effects of these agents in a mouse model and use these systems for new drug design and discovery based on parental compound cuminaldehyde as a lead for safer and potent chemopreventive and/or anticancer usage.
